# Philadelphia Chromosome-Positive Acute Lymphoblastic Leukemia in Children: A Retrospective Cohort Study at a Pediatric Oncology Center

**DOI:** 10.7759/cureus.54154

**Published:** 2024-02-13

**Authors:** Leonardo Maia Moço, Ana Fraga, Iris Maia, Marta Almeida

**Affiliations:** 1 Hematology and Bone Marrow Transplantation, Instituto Português de Oncologia do Porto Francisco Gentil, Porto, PRT; 2 Pediatric Oncology, Instituto Português de Oncologia do Porto Francisco Gentil, Porto, PRT

**Keywords:** allogeneic hematopoietic stem cell transplantation, cranial irradiation, ikaros transcription factor, philadelphia chromosome, precursor b-cell lymphoblastic leukemia-lymphoma

## Abstract

Background and objective

Philadelphia chromosome-positive acute lymphoblastic leukemia (ALL Ph+) is quite rare among pediatric patients. Its management has undergone significant changes in the past few years, leading to some variability in how it is approached. At the Portuguese Oncology Institute of Porto (IPOP), a tertiary oncological center, the standard of care has been aligned with the guidelines proposed by the European intergroup study of post-induction treatment of ALL Ph+ (EsPhALL). In this study, we aimed to examine the experience and outcomes related to the treatment of pediatric patients with ALL Ph+ at IPOP.

Methods

This retrospective cohort study involved pediatric patients diagnosed with ALL Ph+ at IPOP between January 2008 and December 2022 and analyzed their outcomes.

Results

A total of 14 patients were included. *IKFZ1 *was altered in five patients (out of nine in whom it was searched). Five patients were treated according to EsPhALL 2004, which involved starting imatinib later in a discontinuous manner [resulting in both five-year overall survival (OS) and progression-free survival (PFS) of 60%]. The EsPhALL 2010 (preconizing a continuous imatinib regimen instead) was employed in three patients, with a five-year OS and PFS of 66.7%. All children mentioned above received cranial irradiation therapy (CRT). Finally, six were treated according to the EsPhALL 2015, which stopped including CRT in its backbone. The five-year OS was 100%, whereas every patient progressed with an increase in *BCR::ABL1* levels greater than 1-log. Moreover, until 2015, all patients had been recommended to undergo allogeneic hematopoietic stem cell transplantation (alloHSCT). However, since 2015, alloHSCT has been exclusively reserved for relapsed/refractory (R/R) disease or poor responders with positive measurable residual disease (MRD). In total, alloHSCT was performed in nine patients.

Conclusions

Although initially associated with a poor prognosis, the ALL Ph+ paradigm is drastically shifting. Further studies will hopefully clarify the outcomes in this population and help understand the role of central nervous system (CNS) prophylaxis, alloHSCT, and MRD quantification.

## Introduction

Philadelphia chromosome-positive acute lymphoblastic leukemia (ALL Ph+), with the translocation t(9;22)(q34;q11.2), is very rare among the pediatric population, accounting for only 3-5% of acute lymphoblastic leukemias in children [1]. This reciprocal translocation transcripts an aberrant fusion protein, *BCR::ALB1*, whose excessive phosphorylation activity results in increased cell proliferation, differentiation stop, and apoptosis inhibition [2]. The length of the fusion protein depends on the location of the breakpoint. In approximately 66% of ALL Ph+ patients, the breakpoint occurs at the minor bcr region, producing a p190 protein; in the remaining patients, it occurs at the major bcr region, producing a p210 protein. Some studies have reported an association between p190 transcript and a more favorable outcome [3,4].

This disease subtype was classically associated with a poor prognosis, and hence allogeneic hematopoietic stem cell transplantation (alloHSCT) used to be an early option at first remission [1]. However, over the last two decades, the paradigm has radically shifted with the advent of tyrosine kinase inhibitors (TKI) [2,5,6]. Key observations regarding the management of this disease have been drawn by the Children Oncology Group (COG) and the European intergroup study of post-induction treatment of ALL Ph+ (EsPhALL). The latter has conducted a clinical trial (number NCT00287105) [7], which started in 2004 (EsPhALL 2004), and underwent amendments in 2010 (ESPhALL 2010) and 2015 (EsPhALL 2015).

EsPhALL 2010 mainly differed from EsPhALL 2004 in that it advocated for an early introduction of imatinib on day 15, in a continuous regimen for a total of 24 months (compared to a total of three to five months in EsPhALL 2004). Regarding central nervous system (CNS)-directed treatment, cranial irradiation therapy (CRT) would be considered for every patient not eligible for alloHSCT due to the absence of a matched donor (defined as 9/10 or 10/10). This treatment would be placed between two additional re-induction blocks, which in turn would also include lumbar punctures for prophylactic intrathecal methotrexate administration [5,6]. Nevertheless, at the Department of Pediatrics of the Portuguese Oncology Institute of Porto (IPOP), even patients who were candidates for alloHSCT would undergo CRT before it, in an attempt to somehow substitute the aforementioned lumbar punctures. Finally, in 2015, an amendment to the EsPhALL 2010 protocol excluded CRT from its backbone. Moreover, this amendment stipulated that alloHSCT would only be indicated for poor responders with positive measurable residual disease (MRD) instead [7-9].

In this study, our objective is to gain a clearer understanding of how patients at IPOP have been managed under EsPhALL guidelines, as well as to assess their outcomes, thereby laying a strong foundation for future studies on the subject.

## Materials and methods

Study design and setting

We conducted a single-center retrospective cohort study involving patients under the age of 18 years diagnosed with ALL Ph+ at IPOP between January 2008 and December 2022. Data were collected from patients’ clinical records, including demographical, clinical, and molecular characteristics. Treatment protocols were based on the EsPhALL guidelines in use at the respective times. This study was approved by the institutional ethics board of IPOP on 21/12/2023.

Definitions

CNS involvement was classified based on cerebral spinal fluid analysis as follows: "CNS-1" in the absence of blasts on cytospin preparation; "CNS-2" in the presence of <5/µL white blood cells (WBC) and a cytospin positive for blasts; "CNS-3" in the presence of >5/µL WBC and a cytospin positive for blasts; "TLP(-)" if there was a traumatic puncture with no blasts; or "TLP(+)" if there was a traumatic puncture with blasts.

Relapses were classified as follows: “very early” if they occurred less than 18 months after diagnosis; “early” if they occurred 18 months or more after diagnosis but less than six months after the end of treatment; or “late” if they occurred more than six months after the end of treatment. Any increase in BCR::ABL1 quantification by real-time quantitative polymerase chain reaction (RQ-PCR) greater than 1-log was considered to signify a relapsed/refractory disease (R/R).

MRD results are displayed utilizing flow cytometry (sensitivity of 10^-4^), RQ-PCR for *BCR::ABL1* (sensitivity of 10^-5^), and, in some cases, RQ-PCR for immunoglobulin heavy chain and *TCR*-chain gene rearrangements (IG/TR) (sensitivity of 10^-5^) [10,11]. This last methodology was rarely used before 2016, as it required sending samples abroad. After 2016, a protocol with the Portuguese Oncology Institute of Lisbon allowed its implementation as a routine MRD assessment test.

Statistical analysis

For descriptive analysis, qualitative variables are presented as proportions and quantitative variables as medians with the respective interquartile range (IQR). The primary endpoints are overall survival (OS), defined as the time from diagnosis to death from any cause, and progression-free survival (PFS), defined as the time from diagnosis to disease progression or death from any cause. Observational periods were censored at the date of the last contact in case no event was observed. Kaplan-Meier curves were constructed to estimate survival and multivariate Cox regression analysis was used with time to death or progression as the dependent variable and EsPhALL guidelines as the independent variables. All analyses were performed using IBM SPSS Statistics 29.0.0.0 software (IBM Corp., Armonk, NY).

## Results

Baseline characteristics

A total of 14 children were diagnosed with ALL Ph+ at IPOP between January 2008 and December 2022. According to the Epidemiology Service records of the same institute, during that period, 256 children were diagnosed with acute lymphoblastic leukemia. Therefore, ALL Ph+ accounted for 5.5% of all acute lymphoblastic leukemias diagnosed in that timeframe. The main baseline demographic, clinical, and laboratory characteristics of the cohort are displayed in Table 1. The median age was 10 years, and the male-to-female ratio was 1.8:1. Only two children (14%) presented with hyperleukocytosis (defined as a WBC count >100 x 10^9^/L). Four children (29%) displayed a hyperdiploid karyotype, while the others had a diploid or near-diploid karyotype, and no hypodiploidy was identified. Fusion transcript p190 was found in approximately two-thirds of patients (n=9), whereas the remaining one-third had p210 (n=5). IKZF1 alterations, which were only assessed in nine patients starting in 2012, were found in five of them (56%). Only one child presented with gonadal involvement. Three were classified as CNS-2 or CNS-3, although no neurological symptoms were observed.

**Table 1:** Main demographic, clinical, and laboratory characteristics (N=14) AlloHSCT: allogeneic hematopoietic stem cell transplantation; ATG: anti-thymocyte globulin; BM: bone marrow; Bu: Busulfan; CNS: central nervous system; CRT: cranial irradiation; Cy: cyclophosphamide; EsPhALL: European intergroup study of post-induction treatment of Philadelphia-chromosome-positive acute lymphoblastic leukemia; IQR: interquartile range; Mel: Melphalan; R/R: relapsed/refractory disease; TLP: traumatic lumbar puncture; TKI: tyrosine kinase inhibitor; WBC: white blood cell

Variable	Values
Age, years	
Median (IQR)	10 (4.00–13.25)
Male-female ratio	1.8:1
WBC, x 10^9^/L	
Median (IQR)	22.890 (14.255–79.240)
Hyperleukocytosis, n (%)	2 (14%)
Blasts in BM, %	
Median (IQR)	83.85 (72.00–88.25)
Ploidy, n=13, n (%)	
Diploid	7 (53.8%)
Hyperdiploid	4 (30.8%)
Near-diploid	2 (15.4%)
Hypodiploid	0
Fusion transcript, n (%)	
p190	9 (64.3%)
p210	5 (35.7%)
*BCR::ABL1* in BM, %	
Median (IQR)	72.2117 (27.2360–117.9934)
*IKZF1, *n=9, n (%)	5 (56%)
Guidelines, n (%)	
EsPhALL 2004	5 (36%)
EsPhALL 2010	3 (21%)
EsPhALL 2015	6 (43%)
Gonadal disease	1 (7.1%)
CNS disease, n (%)	
CNS-1	8 (57.1%)
CNS-2	1 (7.1%)
CNS-3	2 (14.3%)
TLP(-)	3 (21.4%)
TLP(+)	0
CRT, n (%)	7 (50%)
AlloHSCT, n (%)	9 (64%)
Until 2015, n=8	6
After 2015, n=6	3
Conditioning regimens, n=9, n (%)	
BuCy	1 (11.1%)
BuCyMel	1 (11.1%)
BuCyATG	5 (55.6%)
BuCyMelATG	2 (22.2%)
Donor type, n=9, n (%)	
Matched sibling	2 (22.2%)
Matched unrelated	7 (77.8%)
R/R disease, n (%)	7 (50%)
TKI resistance mutations, n=4, n (%)	1 (25%)
Death, n (%)	3 (21.4%)
Due to infection	1
Due to disease progression	2

Regarding treatment, five children (36%) were treated by following the guidelines proposed by EsPhALL 2004, while nine (64%) were initiated on imatinib on D+15 in a continuous manner. Among the eight children treated based on the guidelines of EsPhALL 2004 and 2010, six underwent alloHSCT at first remission. The remaining two did not have a matched donor. Among the other six children already following the EsPhALL 2015 amendment, three have already undergone alloHSCT. However, this time, only children with MRD positivity would be considered as candidates in the first line. All conditioning regimens were myeloablative and did not include total body irradiation, and all grafts were bone marrow (BM)-derived. None of the transplanted children received post-alloHSCT maintenance with TKI.

Regarding CNS prophylaxis, half of the children received CRT (every child under EsPhALL 2004 and two children out of the three under EsPhALL 2010). The other child (still under EsPhALL 2010 but with a more recent diagnosis) was proposed for alloHSCT but, unlike the others, not for CRT. In fact, EsPhALL preconized that only a few children not suitable for alloHSCT would receive CRT. However, before this patient, it was common practice at the Department of Pediatrics to offer CRT before alloHSCT to every child proposed for it.

**Figure 1:**
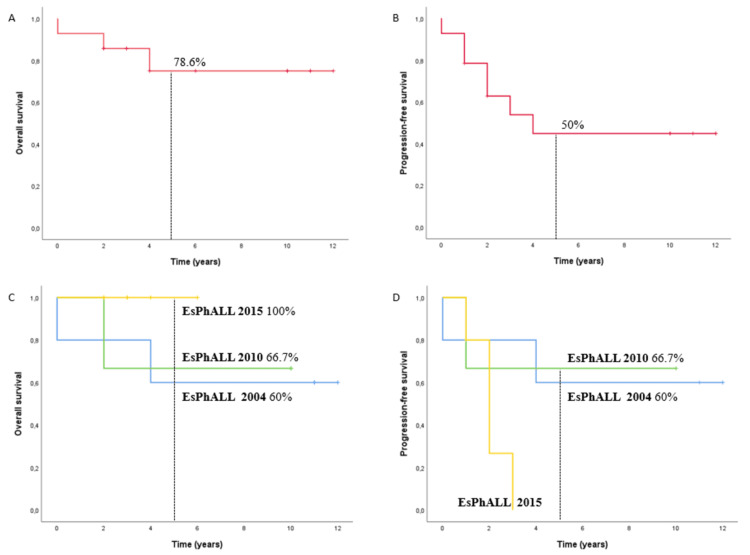
Outcomes in pediatric ALL Ph+ patients Five-year OS (1A) and PFS (1B) in all patients; five-year OS (1C) and PFS (1D) according to the EsPhALL protocol on which practice at IPOP was based ALL Ph+: Philadelphia chromosome-positive acute lymphoblastic leukemia; OS: overall survival; PFS: progression-free survival; EsPhALL: European intergroup study of post-induction treatment of Philadelphia chromosome-positive acute lymphoblastic leukemia

In our cohort, we observed a five-year OS and PFS of 78.6% and 50%, respectively (Figures 1A, 1B). Survival curves stratified by the EsPhALL protocol at use are also depicted (Figures 1C, 1D). Nonetheless, multivariate Cox regression analysis did not show any significant differences between each other. In fact, due to the small sample size, we were unable to determine the impact of baseline characteristics and therapeutic attitudes on patient outcomes.

R/R disease characteristics

Meanwhile, seven patients experienced at least a 1-log increase in *BCR::ABL1 *levels, leading to a second-line treatment option, regardless of their hematological response or flow cytometry results. For study purposes, these children were considered to have R/R disease.

The baseline disease characteristics at the time of diagnosis are detailed in Table 2 and Table 3, for both patients without and with R/R disease, respectively. Among these last set of patients, the characteristics of R/R disease are illustrated in Table 4. Despite the limited sample size, it is interesting to note that every patient with hyperleukocytosis (n=2), CNS-3 disease (n=2), gonadal disease (n=1), and *IKZF1 *alterations (n=5) developed R/R disease. Among the four patients with a hyperdiploid karyotype (linked to better outcomes when the number of chromosomes is greater than 50-67), only one developed R/R disease.

**Table 2:** Baseline characteristics of the patients without R/R disease Blast counts and *BCR::ABL1 *levels were determined in BM samples BM: bone marrow; CNS: central nervous system; Hyper: hyperdiploid; n.a.: not applicable; ND: near-diploid; TLP: traumatic lumbar puncture; WBC: white blood cell

Patient ID	Protocol	WBC, x 10^9^/L	Blasts, %	Ploidy	*BCR::ABL1*,* *%	Transcript	IKZF1	Gonadal disease	CNS
Pt#1	2004	89.80	87.00	ND	178.3750	p210	n.a.	No	1
Pt#2	2004	75.72	66.00	Diploid	311.6040	P210	n.a.	No	1
Pt#3	2004	4.00	33.00	n.a.	37.7610	p190	n.a.	No	1
Pt#4	2004	15.18	83.70	Hyper	128.4351	p190	n.a.	No	1
Pt#5	2010	14.40	84.00	Hyper	21.9291	P190	No	No	TLP(-)
Pt#6	2010	5.96	17.00	ND	18.5711	p190	No	No	TLP(-)
Pt#7	2015	17.39	95.89	Hyper	32.5428	p190	No	No	1

**Table 3:** Baseline characteristics of the seven patients assumed to have R/R disease Blast counts and *BCR::ABL1 *levels were determined in BM samples. BM: bone marrow; CNS: central nervous system; Hyper: hyperdiploid; n.a.: not applicable; ND: near-diploid; TLP: traumatic lumbar puncture; WBC: white blood cell

Patient ID	Protocol	WBC, x 10^9^/L	Blasts, %	Ploidy	*BCR::ABL1*,* *%	Transcript	IKZF1	Gonadal disease	CNS
Pt#8	2015	52.260	93.34	Diploid	107.5517	p210	Yes	Yes	3
Pt#9	2015	19.780	84.82	Diploid	34.7936	p190	Yes	No	1
Pt#10	2015	306.200	78.23	Diploid	72.2117	p210	Yes	No	3
Pt#11	2015	13.820	86.10	Hyper	86.8577	p190	Yes	No	1
Pt#12	2015	504.389	92.00	Diploid	85.5198	p210	Yes	No	1
Pt#13	2010	44.050	74.00	Diploid	16.3224	p190	n.a.	No	2
Pt#14	2004	26.000	79.00	Diploid	n.a.	p190	No	No	TLP(-)

**Table 4:** Characteristics of patients assumed to have R/R disease at the time of switching to second-line therapy MRD was assessed using BM samples *Patient Pt#13, who manifested a second relapse seven months after the first one, with 28.41% of blasts in BM and *BCR::ABL1* levels of 29.8462%. ^+^Patients Pt#13 and Pt#14 who passed away AlloHSCT: allogeneic hematopoietic stem cell transplantation; Blina: blinatumomab; BM: bone marrow; CRT: cranial irradiation; CNS: central nervous system; FC: flow cytometry; IntReALL: International study for treatment of childhood relapsed ALL 2010 version 2.2.; MRD: measurable residual disease

Patient ID	Timing	Location	FC, %	*BCR::ABL1*,* *%	Proposed second-line treatment
Pt#8	Early	BM + CNS	<0.01	0.0108	Dasatinib + CRT + alloHSCT
Pt#9	Very-early	BM	<0.01	0.0137	Dasatinib + alloHSCT
Pt#10	Early	BM	<0.01	8.900	Dasatinib + alloHSCT
Pt#11	Early	BM	<0.01	4.3819	Dasatinib + Blina
Pt#12	Very-early	BM	0.46	4.4127	Dasatinib + Blina + alloHSCT
Pt#13^*+^	Very-early	BM + CNS	62.76	22.1240	IntReALL + Dasatinib
Pt#14^+^	Late	BM + CNS	95.50	22.1933	IntReALL + Imatinib

Only two children (Pt#8 and Pt#9) remained in major molecular response, despite the increase in BCR::ABL1 levels. Nevertheless, they were still proposed for a second-line TKI (dasatinib), followed by alloHSCT. Patient Pt#8 even underwent CRT due to the presence of blast cells in the cerebral spinal fluid. Although proposed for alloHSCT, this child has not undergone the procedure at the time of writing this paper. 

Patients Pt#10 and Pt#11 experienced an isolated loss of molecular response, and hence they were just started on dasatinib. However, while patient Pt#10 proceeded to have alloHSCT, patient Pt#11, who was more recently diagnosed, received concurrent treatment with two cycles of blinatumomab. Patient Pt#12, in addition to losing molecular response, also presented with positive MRD by flow cytometry. Consequently, this patient was proposed for treatment with dasatinib, blinatumomab, plus alloHSCT.

Finally, patients Pt#13 and Pt#14, under the EsPhALL 2010 and 2004 guidelines respectively, resumed induction treatment following the latter protocol IntReALL (International study for treatment of childhood relapsed ALL 2010 version 2.2). Patient Pt#13 was the only patient evolving with R/R disease who had undergone alloHSCT in first-line. Patient Pt#14, although under EsPhALL 2004, did not have a matched donor. Both presented with blasts in the BM aspirate and in the cerebral spinal fluid. Patient Pt#13 even relapsed a second time seven months later, but eventually, both succumbed to disease progression. The third child who passed away had been treated by following the EsPhALL 2004 guidelines. In this case, conversely, death was attributed to an infection and occurred approximately three months after alloHSCT, while in deep molecular response.

Regarding the four patients in whom TKI resistance mutations were searched, only Pt#11 presented the I242T mutation, which is associated with poor sensitivity to imatinib. Additional cytogenetic abnormalities were not screened at the time of relapse.

## Discussion

The rarity of this disease is reflected in our sample size. At IPOP, ALL Ph+ cases accounted for 5.5% of all pediatric acute lymphoblastic leukemias (n=14, out of 256), which approximately aligns with the previously reported proportion of 3-5% [1,12]. ALL Ph+ often presents with *IKZF1 *alterations. In pediatric cases, approximately two-thirds of ALL Ph+ patients exhibit concomitant *IKZF1 *deletions [2]. These abnormalities, in turn, are typically associated with older age at diagnosis, hyperleukocytosis, and higher levels of MRD, all of which are predictors of a poor prognosis [13,14]. In our sample, almost two-thirds of patients displayed *IKZF1 *alterations (n=5, out of a total of 9 in whom those abnormalities were searched), which is quite consistent with the aforementioned findings.

When considering fusion transcript expression and its potential prognostic impact [3,4], within the R/R group, the ratio of p190:p210 expression was around 1:1. Furthermore, among the two patients who succumbed to disease progression, both exhibited p190 protein expression, whereas the patient who passed away due to an infection, free from oncological disease, expressed p210.

As illustrated in this study, the paradigm for ALL Ph+ management has undergone significant changes. The key shifts include (1) initiating TKI therapy earlier; (2) adopting a continuous regimen for TKI; (3) restricting first-line alloHSCT to patients with positive MRD; (4) gradually reducing the use of CRT; and, finally, (5) introducing second-generation TKIs and blinatumomab. Although studies have shown the superiority of dasatinib as a first-line treatment over imatinib, patients at IPOP were managed following the EsPhALL guidelines applicable at the time of diagnosis, with imatinib consistently chosen as the first-line therapy and dasatinib reserved for relapses [15,16]. In cases of morphological relapse, patients were treated based on the IntReALL 2010 in combination with dasatinib [12]. More recently, immunotherapy using blinatumomab, a bispecific antibody targeting CD3 and CD19, has also been recommended for R/R ALL Ph+ alongside a TKI [17-20].

The outcomes in our patients are quite comparable to the ones described in the EsPhALL 2010 report [9]. According to the EsPhALL 2010 report, when compared to EsPhALL 2004, the proportion of patients achieving first complete remission increased from 78% to 97%, and, at five years, the OS and event-free survival were reported as 71.8% and 57.0%, respectively. Similarly, a multicentric Polish study involving 31 pediatric patients under EsPhALL 2010 obtained, at five years, an OS of 74.1% and an even-free survival of 54.2% [21]. Nevertheless, notable toxicity issues were observed [9]. In an attempt to address this main concern around EsPhALL 2010, the patients enrolled in the currently ongoing EsPhALL 2017 trial (number NCT03007147) are receiving imatinib at a dosage of 340 mg/m^2^/day instead of 300 mg/m^2^/day. This trial employs a non-inferiority design to evaluate outcomes in an experimental arm that undergoes a less toxic COG chemotherapy backbone, in comparison with a standard arm receiving a more intensive EsPhALL chemotherapy regimen. Interestingly, CRT has been reintroduced in both arms, but this time it is recommended only for children classified as CNS-3 [22]. This might suggest that the EsPhALL team may have reconsidered the potential importance of CRT in the treatment of these patients. Indeed, while we cannot draw definitive conclusions from these survival curves, there seems to be a trend toward better OS with EsPhALL 2015 guidelines, despite an inferior PFS. This raises questions about the current approaches to these patients, particularly regarding response evaluation, MRD measurement, and, most especially, CNS prophylaxis.

This study has a few limitations, predominantly its small sample size. Furthermore, numerous variables changed between each protocol, making it challenging to draw objective conclusions when comparing them. Additionally, it is important to note that children were not formally enrolled in the EsPhALL trials; instead, the protocols merely served as guidelines for medical practice. For example, patients under the EsPhALL 2004, and two of the three children under the EsPhALL 2010 who underwent alloHSCT, had previously received CRT, despite it not being part of the aforementioned protocols. Moreover, not every patient was screened for *IKZF1 *abnormalities and mutations conferring resistance to TKI, as these procedures were only implemented a few years after the introduction of the EsPhALL protocols.

## Conclusions

This study marks a significant milestone as it is the first of its kind in Portugal to shed light on the clinical practices of a renowned oncological center in the management of pediatric ALL Ph+. Its importance lies not only in the rarity of this condition but also in the rapid evolution of the standard approaches to these patients. Despite substantial advancements observed worldwide, the prognosis remains less than favorable and not fully understood. Therefore, further research is imperative to deepen our understanding of this rare entity, especially concerning response assessment and CNS prophylaxis. We believe this study lays the foundation for a multicenter investigation aimed at providing a comprehensive characterization of the Portuguese experience in this field of expertise.
